# Type I Arabinogalactan and Methyl-Esterified Homogalacturonan Polysaccharides from Tamarillo (*Solanum betaceum* cav.) Fruit Pulp Ameliorate DSS-Induced Ulcerative Colitis

**DOI:** 10.3390/ph18040461

**Published:** 2025-03-25

**Authors:** Lara Luisa Valerio de Mello Braga, Carolina Silva Schiebel, Gisele Simão, Karien Sauruk da Silva, Mateus Henrique dos Santos Maia, Ana Carolina Vieira Ulysséa Fernardes, Georgia E. do Nascimento, Lucimara Mach Côrtes Cordeiro, Tufik Adel Issa, Marcelo Biondaro Gois, Elizabeth Fernandes Soares, Daniele Maria-Ferreira

**Affiliations:** 1 Instituto de Pesquisa Pelé Pequeno Príncipe, Faculdades Pequeno Príncipe, Curitiba 80250-060, PR, Brazil; laraluisabraga1@gmail.com (L.L.V.d.M.B.); carolschiebel@hotmail.com (C.S.S.); gisele_si@hotmail.com (G.S.); kariensauruk@outlook.com (K.S.d.S.); mateus.maia@aluno.fpp.edu.br (M.H.d.S.M.); lizbeth_fernandes@yahoo.co.uk (E.F.S.); 2 Programa de Pós-graduação em Biotecnologia Aplicada à Saúde da Criança e do Adolescente, Faculdades Pequeno Príncipe, Curitiba 80230-020, PR, Brazil; 3 Departamento de Bioquímica e Biologia Molecular, Universidade Federal do Paraná, Curitiba 81531-980, PR, Brazillucimaramcc@ufpr.br (L.M.C.C.); 4 Faculdade de Ciências da Saúde, Universidade Federal de Rondonópolis, Rondonópolis 78736-900, MT, Brazilmarcelobiondaro@gmail.com (M.B.G.)

**Keywords:** ulcerative colitis, inflammatory bowel disease, polysaccharides, tamarillo

## Abstract

**Background:** Inflammatory bowel diseases, such as ulcerative colitis and Crohn’s disease, affect the gastrointestinal tract. Treatment aims to induce remission and relieve symptoms but may fail or cause side effects. Recent studies suggest that natural polysaccharides can reduce inflammation and promote healing. The polysaccharides of the pulp of tamarillo (*Solanum betaceum* cav.) have shown beneficial effects, but their potential in colitis is still unexplored. **Objective:** To investigate the effect of polysaccharides from tamarillo pulp in an animal model of ulcerative colitis. **Methods**: Polysaccharides from tamarillo pulp (STWA) were extracted and tested in female mice (BALB/c) to investigate their effect on dextran sodium sulfate (DSS)-induced ulcerative colitis. Different doses of the polysaccharides were tested (10 mg/kg, 30 mg/kg, and 100 mg/kg). The course of the disease and the weight of the animals were monitored daily. At the end of the experimental protocol, the large intestine was removed and measured. Markers of oxidative stress and inflammation were then analyzed. Histological analysis was performed to assess microscopic changes. **Results**: Treatment with STWA (100 mg/kg) prevented weight loss in mice with DSS-induced colitis and reduced the disease activity index. The colon length was preserved, and occult blood in the feces was reduced. Treatment with STWA controlled oxidative stress. Glutathione S-transferase (GST) levels increased, while lipid peroxidation decreased. The inflammatory process was reduced, as indicated by the decrease in myeloperoxidase (MPO), N-acetylglucosamine (NAG), and tumor necrosis factor alpha (TNF-α) levels and the increase in interleukin 10 (IL-10) levels. STWA also improved the colon histology, while preserving the colonic epithelium. **Conclusions:** The results suggest that STWA has protective potential and reduces inflammation in an experimental model of ulcerative colitis in mice.

## 1. Introduction

Inflammatory bowel disease (IBD) includes both acute and chronic inflammation of the digestive tract, with ulcerative colitis (UC) and Crohn’s disease (CD) being the most well-known types [1]. The exact cause of IBD is still nuclear [2,3], but it is believed to result from a combination of genetic factors, immune system dysfunction, problems with the gut barrier, and environmental influences such as infections, changes in gut microbiota, and other unknown triggers [4,5,6,7].

UC is a progressive disease that can lead to persistent inflammation of the colon and rectum mucosa [8]. In severe cases, the inflammatory process can extend to the submucosa and affect the entire colon [9]. The disease affects people of all ages [10], with bloody diarrhea and tenesmus being the main symptoms [4]. The primary therapeutic goal of UC is to induce remission of the disease, which includes symptom relief, mucosal healing, and long-term remission [11,12]. Achieving these goals should improve patients’ quality of life and reduce the need for hospitalization and colectomies [13,14]. Standard treatment for UC includes 5-aminosalicylic acid (5-ASA), corticosteroids, and thiopurines. However, not all drugs are effective for both induction and maintenance of remission, and there is increasing evidence of potentially serious adverse effects associated with their use [12].

Pharmacologic treatment of UC has evolved significantly over the past few decades, and new treatment options such as anti-tumor necrosis factor (anti-TNF), anti-adhesion agents, anti-IL-12/interleukin-23p40 (anti-IL-12/IL-23p40), and Janus kinase (JAK) inhibitors have been introduced. These advances have effectively improved the prognosis of patients [15]. However, these therapeutic approaches are associated with significant challenges, including treatment failure, serious adverse effects, such as the development of antibodies against the drug, and a significant financial burden [15,16,17,18].

There is increasing evidence for the central role of dietary components in influencing inflammatory processes associated with ulcerative colitis (UC) [19]. Among these, natural polysaccharides have gained increasing attention due to their versatile therapeutic potential. Natural polysaccharides have demonstrated the potential to alleviate UC-related inflammation by reducing inflammatory mediators, restoring the balance of the gut microbiota, and improving the integrity of the intestinal barrier [20,21,22,23,24].

In this study, we investigated the potential bioactivity of type I arabinogalactan and highly methyl-esterified homogalacturonan polysaccharides from tamarillo fruit pulp (*Solanum betaceum* cav.) in a model of DSS-induced experimental ulcerative colitis. The tamarillo, a fruit native to the Andean region, is not only known for its high nutritional value, but also for its bioactive compounds that support its use in traditional medicine [25]. The pectin polysaccharides contained in the pulp of the tamarillo fruit have been studied and have shown remarkable antinociceptive and anti-inflammatory properties [26]. In addition, pectic polysaccharides, such as arabinogalactans and homogalacturonans, derived from various sources, have demonstrated efficacy in alleviating experimental ulcerative colitis, for example, by reducing pro-inflammatory cytokines. These bioactive polysaccharides exert their therapeutic effect by protecting the epithelial layer—strengthening junction proteins, attenuating inflammatory responses, and promoting the reorganization of the intestinal microbiota [27,28,29,30]. However, the effect of tamarillo arabinogalactan type I and methyl-esterified homogalacturonan polysaccharides in the treatment of colitis is not known. In this study, it was hypothesized that pectic polysaccharides derived from tamarillo could alleviate ulcerative colitis by reducing intestinal inflammation and restoring intestinal homeostasis.

## 2. Results

### 2.1. Treatment with STWA Attenuates DSS-Induced Colitis

**Figure 1:**
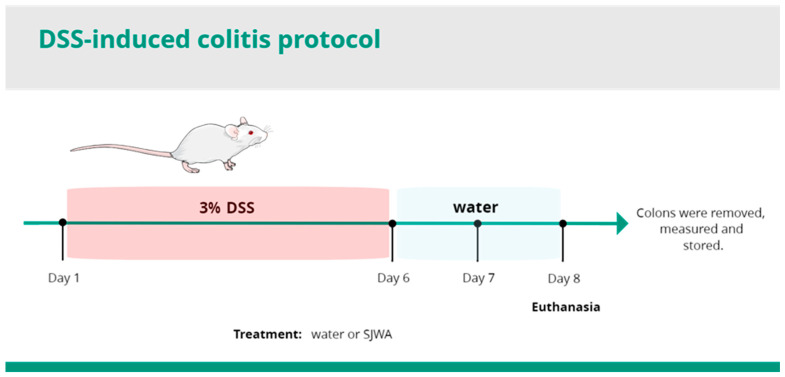
Experimental model for ulcerative colitis.

Mice were treated orally with STWA (10, 30, and 100 mg/kg) or vehicle (water, 1 mL/kg) once daily from day 1 to day 8 to investigate its potential effect on DSS-induced ulcerative colitis (Figure 1). Body weight fluctuations and disease activity index (DAI) were determined daily according to the experimental protocol.

**Figure 2:**
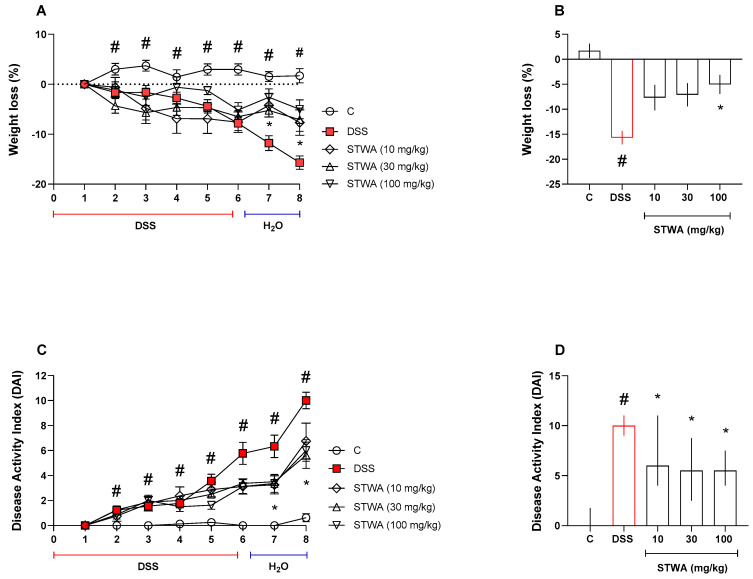
Effect of STWA on (**A**,**B**) body weight changes and (**C**,**D**) disease activity index. The mice were administered 3% DSS in drinking water for 5 consecutive days, followed by 2 days of water. Mice were treated orally with vehicle (water, 1 mL/kg) or STWA (10, 30 or 100 mg/kg) once daily for 8 days. Results are expressed as mean ± SEM or median and interquartile range and were analyzed by two-way ANOVA (**A**,**C**) followed by Bonferroni test for multiple comparisons, or Kruskal–Wallis followed by Dunn’s test (**B**,**D**). # *p* < 0.05, compared to the control group; * *p* < 0.05, compared to the DSS group. n = 8–12 animals per group.

Mice that received DSS in their drinking water and were treated with the vehicle began to lose weight on day 2 (−1.17%, *p* = 0.0444). The weight loss persisted until day 8 of the experimental protocol (−15.67%, *p* < 0.0001) compared to the control group (Figure 2A,B). The DSS group had an increased disease activity index (DAI) from day 2 (median: 1) to day 8 (median: 10) compared to the control group (Figure 2C,D).

**Figure 3:**
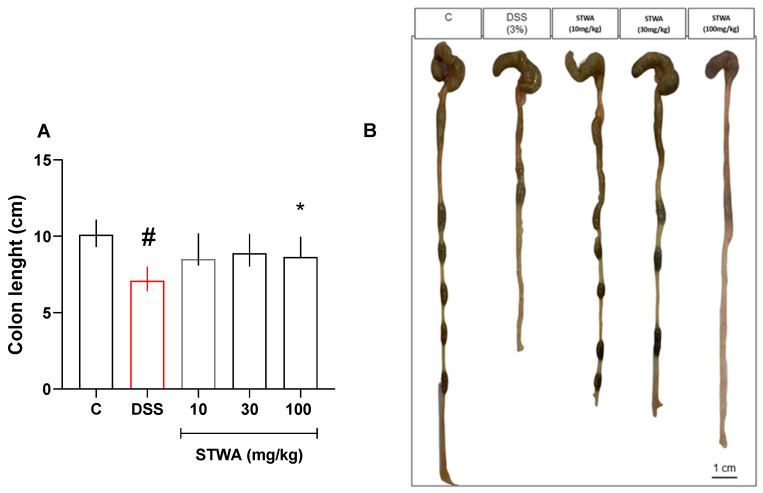
Effect of STWA on the length of the colon (**A**,**B**). The mice were given 3% DSS in drinking water for 5 consecutive days, followed by 2 days of water. Mice were treated orally with vehicle (water, 1 mL/kg) or STWA (10, 30 or 100 mg/kg) once daily for 8 days. Results are expressed as median and interquartile range and were analyzed by Kruskal–Wallis followed by Dunn’s test (**A**). # *p* < 0.05, compared to the control group; * *p* < 0.05, compared to the DSS group. n = 8–12 animals per group.

The animals in the DSS group showed occult blood in the stool from day 3 of the protocol. In contrast, the animals in the control group showed no occult blood in the stool (Table 1). In addition, the length of the colon was reduced in the DSS group (median: 7.1 cm) compared to the control group (median: 10.1 cm) (Figure 3A,B).

Administration of STWA (100 mg/kg) significantly prevented body weight loss (day 7: −5.24%; day 8: −7.08%) (Interaction [F (28,252) = 4.733]; Time [F (3.761,135.4) = 16.49]; *p* < 0.0001) (Figure 2A,B) and the development of DAI (day 7, median: 3.37; day 8, median: 5.62) (Interaction [F (28,252) = 5.917]; Time [F (2.811,101.2) = 66.22]; *p* < 0.0001) compared to the DSS group (Figure 2C,D).

The positivity of occult blood also decreased, with approximately 50% of the animals showing no occult blood in the stool compared to the DSS group (Table 1). Treatment with STWA prevented shortening of the colon at the 100 mg/kg dose (median: 8.65) compared to the DSS group (Figure 3A,B).

### 2.2. STWA Reduces Oxidative Stress

**Figure 4:**
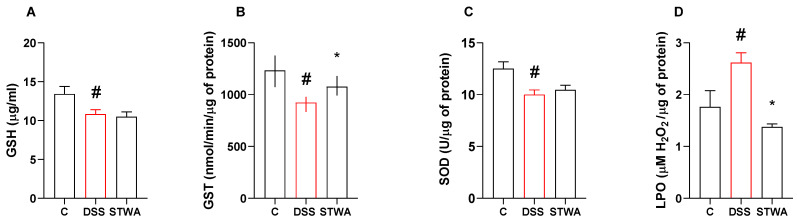
Effect of STWA on GSH levels (**A**), GST (**B**) and SOD activity (**C**), and LPO quantification (**D**). The mice were administered 3% DSS in drinking water for 5 consecutive days, followed by 2 days of water. The mice were treated orally with vehicle (water, 1 mL/kg) or STWA (100 mg/kg) once daily for 8 days. The results are expressed as a mean ± SEM or a median and interquartile range and were analyzed by one-way ANOVA followed by the Bonferroni test for multiple comparisons (**A**,**C**,**D**), or Kruskal–Wallis followed by Dunn’s test (**B**). # *p* < 0.05, compared to the control group; * *p* < 0.05, compared to the DSS group. n = 6–12 animals per group.

Treatment with DSS reduced GSH levels in the DSS group (10.8 ± 0.5 µg/mL, *p* = 0.0436) compared to the control group (13.42 ± 0.97 µg/mL, *p* = 0.0436) ([F (2,21) = 4.626]; *p* = 0.0216). Treatment with STWA did not reverse this result (Figure 4A).

DSS treatment decreased GST activity in the colon (median: 923.46 nmol/min/μg protein) compared to the control group (median: 1233 nmol/min/μg protein). STWA at a dose of 100 mg/kg prevented the decrease in GST activity (median: 1077.00 nmol/min/μg protein) compared to the DSS group (Figure 4B).

SOD activity was reduced after the DSS protocol (10.01 ± 0.43 U/μg protein), compared to the control group (12.51 ± 0.66 U/μg protein, *p* = 0.0060) ([F (2,19) = 6.324]; *p* = 0.0078) (Figure 4C). Treatment with STWA could not prevent this decrease.

LPO levels were increased by DSS treatment (2.61 ± 0.19 μM H_2_O_2_/μg protein) compared to the control treated group (1.76 ± 0.31 μM H_2_O_2_/μg protein, *p* = 0.0393). The STWA- treated group showed a reduction in LPO levels compared to the DSS group (1.37 ± 0.05 μM H_2_O_2_/μg protein, *p* = 0.0034) ([F (2,31) = 6.887]; *p* = 0.0034) (Figure 4D).

### 2.3. Effect of STWA on Inflammatory Parameters

**Figure 5:**
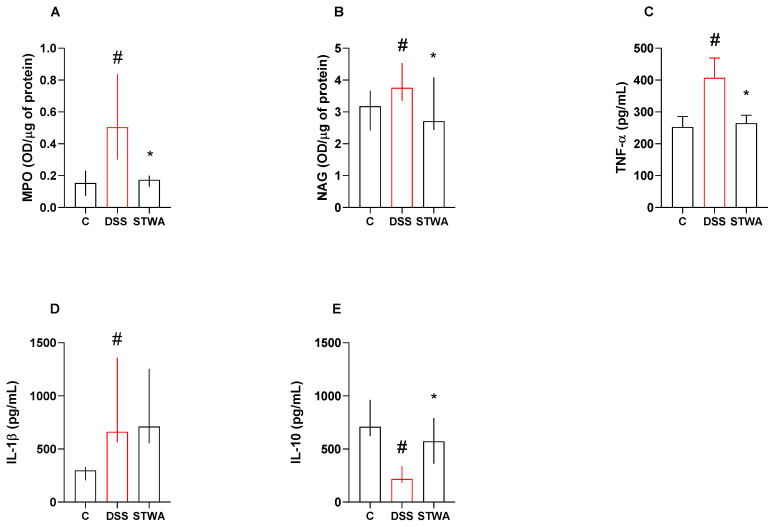
Effect of STWA on MPO (**A**) and NAG (**B**), TNF-α (**C**), IL-1β (**D**), and IL-10 (**E**) levels. The mice were administered 3% DSS in drinking water for 5 consecutive days, followed by 2 days of water. The mice were treated orally with vehicle (water, 1 mL/kg) or STWA (100 mg/kg) once daily for 8 days. The results are expressed as a mean ± SEM or a median and interquartile range and were analyzed by one-way ANOVA followed by the Bonferroni test for multiple comparisons (**C**), or Kruskal–Wallis followed by Dunn’s test (**A**,**B**,**D**,**E**). # *p* < 0.05, compared to the control group; * *p* < 0.05, compared to the DSS group. n = 8–12 animals per group.

MPO activity was increased after the DSS protocol (median: 0.50 OD/μg protein) compared to the control group (median: 0.15 OD/μg protein). STWA at a dose of 100 mg/kg decreased MPO activity (median: 0.17 OD/μg protein) compared to the DSS group (*p* = 0.0443) (Figure 5A). Treatment with DSS also increased NAG activity (median: 3.75 OD/μg protein) compared to the control group (3.17 OD/μg protein). STWA (100 mg/kg) decreased NAG levels (median: 2.70 OD/μg protein) compared to the DSS group (Figure 5B).

TNF-α levels increased after the DSS protocol (407.10 ± 61.75 pg/mg) compared to the control (252.60 ± 33.06 pg/mg; *p* = 0.0299). However, treatment with STWA reduced TNF-α levels compared to the DSS group (264.50 ± 25.26 pg/mg; *p* = 0.0310) ([F (2,20) = 4.459]; *p* = 0.0250) (Figure 5C).

IL-1β levels were also increased after the DSS protocol (median: 661.30 pg/mg) compared to control (median: 298.40 pg/mg). Treatment with STWA could not prevent this increase (median: 710.00 pg/mg) (Figure 5D). On the other hand, IL-10 levels decreased after the DSS protocol (median: 218.50 pg/mg) compared to control (median: 709.8 pg/mg), and treatment with STWA prevented this decrease (median: 571.40 pg/mg).

### 2.4. Effect of STWA on Histological Parameters

**Figure 6:**
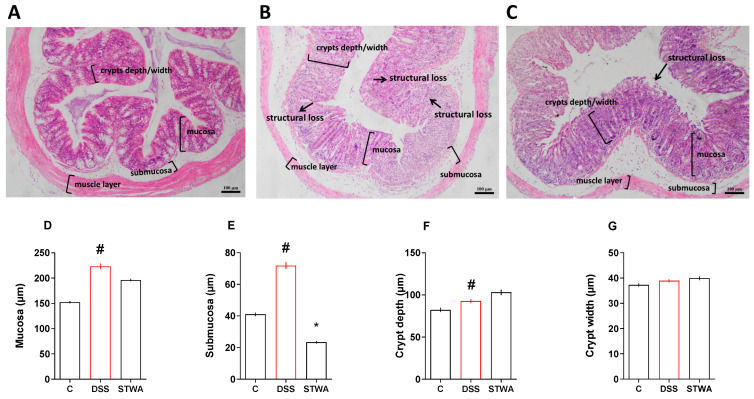
Effect of STWA on histological parameters. Alcian blue-stained sections of colons, ×10 magnification (bars = 100 μm) from the control group (**A**), DSS group (**B**), and STWA group (**C**) showing the mucosa, submucosa, and muscle layer with markers for existing structural loss and depth/width of crypt. The data from the images were quantified and presented in graphs regarding the change in mucosa (**D**), submucosa (**E**), crypt depth (**F**), and crypt width (**G**). The mice were administered 3% DSS in drinking water for 5 consecutive days, followed by 2 days of water. The mice were treated orally with vehicle (water, 1 mL/kg) or STWA (100 mg/kg) once daily for 8 days. The results are expressed as a median and interquartile range and were analyzed by Kruskal–Wallis followed by Dunn’s test (**D**–**G**), # *p* < 0.05, compared to the control group; * *p* < 0.05, compared to the DSS group. There were 4–6 animals per group.

The histomorphological analyses showed significant differences between the groups. Treatment with DSS increased the thickness of the mucosa (µm) (median: 212.4) (Figure 6D), the submucosa (µm) (median: 61.8) (Figure 6E), and the depth of the crypt (µm) (median: 83.8); (Figure 6F) compared to the vehicle group (median: 157.42; 38.54 and 71.99, respectively). No differences were observed in the measurement of crypt width (µm) (Figure 6G). The administration of STWA (100 mg/kg) improved the histomorphometry of the colon in the submucosa (median: 21.78) compared to the DSS group (median: 61.8) (Figure 6E).

**Figure 7:**
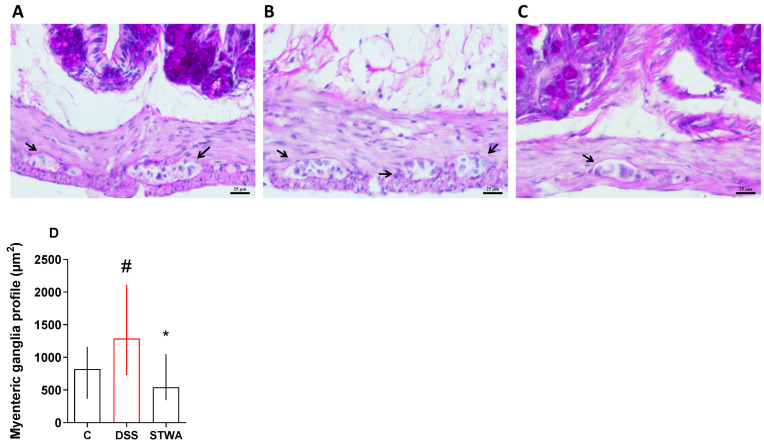
Effect of STWA on the profile of the myenteric ganglia. Periodic Acid Schiff-stained sections of the colon, ×20 magnification (bar = 25 μm) from the control group (**A**), the DSS group (**B**), and the STWA group (**C**), showing the difference from the myenteric ganglia profile, marked by the arrow. These data are quantified in graph (**D**). The mice received 3% DSS in drinking water for 5 consecutive days, followed by 2 days of water. The mice were treated orally with vehicle (water, 1 mL/kg) or STWA (100 mg/kg) once daily for 8 days. The results are expressed as a median and interquartile range and were analyzed by Kruskal–Wallis followed by Dunn’s test (**D**), # *p* < 0.05, compared to the control group; * *p* < 0.05, compared to the DSS group. There were 4–6 animals per group.

Histomorphometric analysis revealed that treatment with DSS resulted in a significant increase in the myenteric ganglia profile area (Figure 7), with a median area of 1287.22 µm^2^ compared to the control group, which had a median value of 820.35 µm^2^. In contrast, the administration of STWA (100 mg/kg) resulted in a reduction in the profile of the myenteric ganglia to a median value of 541.46 µm^2^ (Figure 7D).

**Figure 8:**
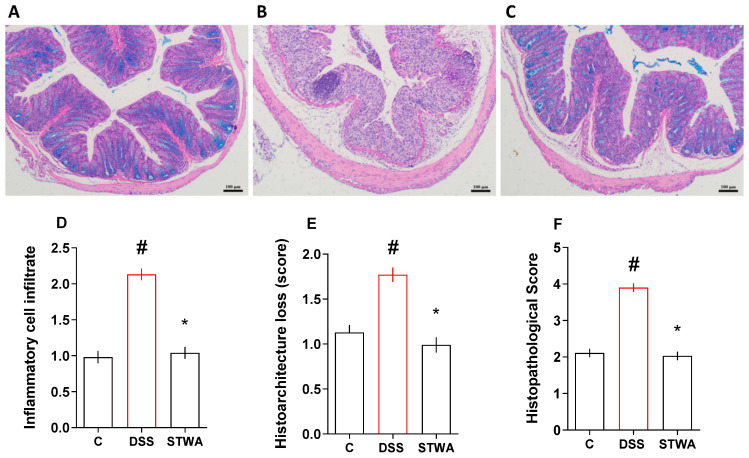
Effect of STWA on Alcian blue and PAS-stained sections of colons, ×20 magnification (bars = 100 μm) from the control group (**A**), DSS group (**B**), and STWA group (**C**). Inflammatory cellular infiltrate count (**D**), Histoarchitectural loss (**E**) and histopathological score (**F**). The mice were administered 3% DSS in drinking water for 5 consecutive days, followed by 2 days of water. The mice were treated orally with vehicle (water, 1 mL/kg) or STWA (100 mg/kg) once daily for 8 days. The results are expressed as a median and interquartile range and were analyzed by Kruskal–Wallis followed by Dunn’s test (**A**–**C**), # *p* < 0.05, compared to the control group; * *p* < 0.05, compared to the DSS group. n = 4–6 animals per group.

Treatment with DSS caused an increase in inflammatory cells (median: 2) compared to the control group (median: 1), which was prevented by treatment with 100 mg/kg STWA (median:1) compared to the diseased group (Figure 8D). The histoarchitecture of the colon was also calculated (Figure 8E). DSS treatment increased the loss of histoarchitecture (median: 2) when compared to control (median: 1), which was prevented by treatment with STWA treatment (median: 1). Finally, treatment of the group with DSS increased the histopathologic score (median: 4) (Figure 8F) compared to the control (median: 2), which was prevented by treatment with STWA (median: 2). Figure 8A–C represent the control, DSS, and STWA groups, respectively, stained with PAS and Alcian blue.

### 2.5. PAS and Alcian Blue Staining Show Mucosal Recovery with STWA

**Figure 9:**
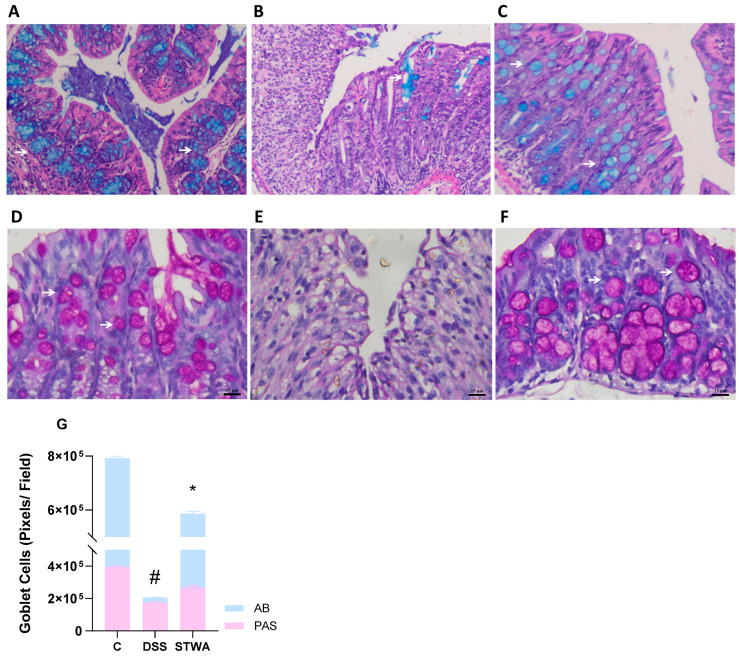
Effect of STWA on histochemical staining for acidic mucin (Alcian blue) of the control group (**A**), the DSS group (**B**) and the STWA group (**C**), and neutral mucin-like glycoproteins (PAS) of the control group (**D**), the DSS group (**E**), and the STWA group (**F**). Histochemical staining of the colon for neutral mucin-like glycoproteins (PAS) and acidic mucin (Alcian blue), ×20 (bar = 15 μm), and the graphic representation of the goblet cell counts in both stains (**G**). The white arrows show the accumulation of mucus in the tissue. The mice were administered 3% DSS in drinking water for 5 consecutive days, followed by 2 days of water. The mice were treated orally with vehicle (water, 1 mL/kg) or STWA (100 mg/kg) once daily for 8 days. The results are expressed as a median and interquartile range and were analyzed by Kruskal–Wallis followed by Dunn’s test (**G**), # *p* < 0.05, compared to the control group; * *p* < 0.05, compared to the DSS group. There were 4–6 animals per group.

The histological images with PAS and Alcian blue staining show clear differences between the groups (Figure 9). Alcian blue staining shows notable changes between the groups. In the control group (Figure 9A), Alcian blue staining highlights a well-organized presence of acidic mucins. In contrast, the DSS group showed reduced Alcian blue staining (Figure 9B), indicating mucosal damage.

In contrast, the STWA group showed a more uniform Alcian blue staining pattern (Figure 9C), indicating an improvement in the mucous membranes. In the control group (Figure 9D), the PAS staining showed a uniform mucosa with intense pink coloration, indicating healthy glycoprotein content. In contrast, the DSS group (Figure 9E) showed a thickened mucosa with irregular staining. The STWA group showed improved mucosa with more uniform PAS staining (Figure 9F). Treatment with DSS significantly reduced the number of goblet cells compared to the control group in PAS staining (221,170 ± 10,925, *p* < 0.0001). However, STWA treatment prevented this depletion (PAS: 92.854 ± 10.925, *p* < 0.0001) ([F(2,1146) = 84.98]; *p* < 0.0001) compared with the DSS group (Figure 9G). Similarly, DSS treatment resulted in a significant reduction in goblet cells, as shown by the lower number of AB staining (364.470 ± 10.925, *p* < 0.0001). STWA treatment attenuated this decrease (AB: 287,099 ± 10,925, *p* < 0.0001) ([F (2,1146) = 84.98]; *p* < 0.0001) compared to the DSS group (Figure 9G).

## 3. Discussion

Natural bioactive compounds are increasingly being used as complementary therapies for intestinal inflammation. These include plant polysaccharides, which improve the integrity of the intestinal epithelium, reduce inflammation, and modulate the intestinal microbiota [31]. Polysaccharides with different structural configurations, such as arabinogalactans, rhamnogalacturonans, homogalacturonans, and β-glucans, have demonstrated their efficacy in alleviating symptoms in experimentally induced colitis models [32,33,34,35]. The pectic polysaccharides of the pulp of the tamarillo fruit, containing type I arabinogalactan and methyl-esterified homogalacturonan have already been investigated and showed promising biological activity. In this study, we have shown that it can effectively alleviate intestinal inflammation in an experimental model of ulcerative colitis.

The DSS colitis model is commonly used to study UC because it directly damages the epithelial cells of the colon, disrupts the integrity of the epithelium, and increases permeability [36], ultimately leading to weight loss, diarrhea, and rectal bleeding [37]. Increased intestinal permeability facilitates the penetration of antigens and bacteria into the intestine, which triggers an inflammatory response mediated primarily by the innate immune system [38,39]. This response is mainly driven by macrophages and neutrophils, which secrete pro-inflammatory cytokines such as TNF-α, IL-1β, and IL-6, leading to persistent inflammation and tissue damage [40,41].

The results of this study are consistent with the experimental model. The administration of DSS successfully induced UC in mice, as evidenced by weight loss, increased disease score, and shortening of the colon. The administration of STWA reversed weight loss and rectal bleeding, improved stool consistency, and lowered DAI score. In addition, STWA prevented the shortening of the colon, which indirectly indicates a reduction in the inflammatory process. Other researchers who have investigated the protective potential of polysaccharides have also demonstrated attenuation of the early aspects of experimental UC. Polysaccharides, such as arabinogalactans and homogalacturonans, have been shown to attenuate the pathologic symptoms of experimental ulcerative colitis, including weight loss, increased disease activity index, shortening of the colon, and damage to intestinal and splenic tissues [22,23,28,29].

Excessive inflammatory reactions contribute decisively to the destruction of the intestinal barrier in UC [42]. Activation of antigen-presenting cells facilitates the differentiation of naïve CD4+ T cells into Th-2 effector cells, leading to the production of various pro-inflammatory cytokines, including TNF-α, IL-1β, IL- 6, and IL-13 [42,43,44,45,46]. The overproduction of pro-inflammatory cytokines in conjunction with a defective regulatory immune response leads to persistent inflammation that contributes to severe and permanent damage to the epithelium and accelerates the progression of UC [11,47]. In addition, oxidative stress has long been recognized as a key factor in the development and progression of ulcerative colitis [48,49]. In this context, epithelial cells, neutrophils, and macrophages contribute significantly to the production of reactive oxygen and nitrogen species, including superoxide and nitric oxide. This production is mediated by the activation of NADPH oxidase and inducible nitric oxide synthase (iNOS), both of which are upregulated by inflammatory cytokines. The resulting oxidative stress not only damages the intestinal epithelium but also exacerbates the inflammatory response [50,51]. Consequently, the above factors are considered critical indicators of disease severity, and therapeutic strategies aimed at attenuating inflammation and oxidative stress have the potential to delay disease progression and ameliorate disease outcomes. STWA helped to prevent the development of an intense inflammatory process, as evidenced by a reduction in MPO, NAG (indirect markers for the assessment of neutrophil and macrophage infiltration), and TNF-α levels and an increase in IL-10 levels compared to the DSS group. STWA also reduced lipid peroxidation and GST levels, indicating a reduction in the inflammatory process and oxidative stress.

Accordingly, Zhang et al. [23] demonstrated that a food-derived arabinogalactan attenuated the secretion of the pro-inflammatory cytokines TNF-α and IL-1β and promoted the release of IL-10 in a DSS-induced acute colitis model. It also reduced MPO activity in intestinal tissue. The authors also showed that the polysaccharide-modulated key signaling pathways, including the NF-κB, MAPK, and PPARγ signaling pathways, inhibited the NLRP3 inflammasome signaling pathway, resulting in a reduction in DSS-induced colitis inflammation. Another arabinogalactan from the medicinal and edible plant Ixeris *chinensis* (Thunb.) Nakai showed immunomodulatory activity, as evidenced by inhibition of TNF-α, IL-1β, and NLRP3 overproduction and IL-10 secretion in colon tissue [29]. Similarly, a homogalacturonan-enriched pectin-based hydrogel alleviated the effects of colitis by reducing inflammation via the NF-κB/NLRP3 axis [28].

Improving the integrity of the intestinal protective barrier is a promising strategy to mitigate disease progression. This approach relies on the crucial role of the protective mucus layer, goblet cells, and the expression of mucins, which together contribute to the maintenance of an intact epithelial barrier [52,53,54]. Microscopic analysis of the colon showed that DSS caused significant histologic changes, with a loss of tissue architecture, a reduction in the protective mucus layer, depletion of goblet cells, and infiltration of inflammatory cells. Previous studies have also shown that pectin polysaccharides have the potential to improve the barrier of the intestinal epithelium both in vitro and in vivo. Maintaining barrier function helps to restore intestinal integrity and maintain homeostasis [21,55,56].

In accordance with these results, treatment with STWA preserved the epithelial architecture. Histologic analyses showed a reduction in the inflammatory infiltrate and histopathologic score. Importantly, STWA preserved the epithelial architecture of the intestine. The results confirm the preservation of mucin-positive staining and goblet cells. Therefore, we can hypothesize that STWA protects the mucosal barrier, maintains the healthy colonic epithelium and thus contributes to the prevention of the exacerbated inflammatory process, culminating in the reduction in the overall aspects of experimental UC. Maintaining the profile of the myenteric ganglia could also help to reduce the diarrhea-like aspect of the stool observed after treatment with STWA.

Our results show that STWA effectively reduces inflammation and oxidative stress while repairing the damage to the intestinal barrier caused by DSS. Even if the exact mechanism of action of STWA has not yet been researched, it is important to know that polysaccharides are complex molecules that can exert their effect via a number of direct and indirect mechanisms [57,58]. Polysaccharides can regulate signaling pathways [33], control the release of cytokines [59], improve immune function and reduce oxidative stress [60], maintain the homeostasis of the intestinal environment [61], and protect the colonic mucosa [56]. Certain structures can interact directly with intestinal epithelial receptors, including Toll-like receptors [62,63] and mucins via molecular mucoadhesive interactions [64,65,66], which may help to maintain the mucus layer and support the epithelial barrier while reducing local inflammation. The protective effect of polysaccharides is also linked to their fermentation by gut microbiota, which results in the production of short-chain fatty acids (SCFAs) and other metabolites. These metabolites activate specific G-protein-coupled receptors, such as GPR41, GPR43, and GPR109A [67,68]. Polysaccharides can also stimulate the growth of gut bacteria, leading to alterations in the composition of the gut microbiota and, consequently, influencing the overall health of the host [67]. These mechanisms should be further investigated. Nevertheless, our study improves the understanding of the functions of STWA and provides a theoretical basis for the use of plant polysaccharides as adjuvant therapy in ulcerative colitis.

## 4. Materials and Methods

### 4.1. Pectic Polysaccharides from Tamarillo Fruit Pulp

The fraction (named STWA) containing the pectic polysaccharides from tamarillo fruit investigated in this study was previously extracted and chemically characterized by Do Nascimento et al. (2015). It consists mainly of Ara (29.6%), Gal (28.5%), and GalA (30.4%), with minor amounts of Rha, Xyl, Man, and Glc. NMR analysis showed that STWA contains a type I arabinogalactan (AGI) and a highly methyl-esterified (DE = 71%) homogalacturonan.

A voucher specimen of the plant was deposited in the herbarium of the Department of Botany of the Federal University of Paraná (UFPR) under registration number 72,896 and the access was registered with the National System for Managing Genetic Heritage and Associated Traditional Knowledge under the number AB27934.

### 4.2. Animals

Female BALB/c mice aged 6 to 8 weeks and weighing between 20 and 25 g were used for the study. The animals were provided by the animal facility of the Instituto Carlos Chagas, Fiocruz, Curitiba, Paraná, Brazil. The animals were housed in plastic cages with environmental enrichment during the acclimatization period and throughout the experimental protocols, with a maximum of 12 animals per cage. The cages were lined with wood shavings and the animals were acclimatized under controlled conditions of temperature (23 ± 2 °C), humidity (60 ± 10%), and lighting (12 h light-dark cycle) with free access to water and food. The bedding and environmental enrichment were changed every three days. In addition, the animals were acclimatized to the environment of the animal facility and the experimenters two weeks before the start of the experiments. For the experimental protocol, the animals were randomly divided, matched according to their weight and labeled according to their respective experimental group. All procedures were performed in the morning to ensure consistency. The experimental protocols were submitted to the Ethics Committee for the Use of Animals (CEUA) of the Pequeno Príncipe Research Institute and approved under number—069/2022 (approved on 20 October 2022). All procedures performed in this study were in strict compliance with the ethical principles of the Brazilian College of Animal Experimentation (COBEA) and the guidelines of the National Council for the Control of Animal Experimentation (CONCEA) and ARRIVE (https://arriveguidelines.org/). All researchers involved in the project received technical training in the handling of laboratory animals and were trained by an experienced experimenter before starting the activities.

### 4.3. Induction and Evaluation of DSS Ulcerative Colitis

Acute ulcerative colitis was induced with 3% DSS (dextran sodium sulfate, molecular weight: 40,000, Cayman Chemical Company, Ann Arbor, MI, USA). DSS was diluted in drinking water and offered to the animals on 6 consecutive days. On days 7 and 8 of the experimental protocol, the DSS was replaced with normal drinking water (Figure 1). The clinical course of the disease (Disease Activity Index: DAI), which included loss of body weight, stool consistency, and visible blood in the stool, was monitored throughout the experimental period. Occult blood was measured separately. On the eighth day, all animals were euthanized. The colon was carefully removed, thoroughly rinsed with 0.9% saline, measured, and immediately stored at −80 °C or 4% formaldehyde at room temperature for later analysis.

### 4.4. Pharmacological Treatment and DAI Measurement

To evaluate the protective effect of STWA, animals were subjected to the following treatments: (i) control group treated with vehicle (water) (control: 0.1 mL/10 g, [p.o.]); (ii) DSS group treated with vehicle and receiving DSS in drinking water (DSS: water, 0.1 mL/10 g, [p.o.]); and (iii) STWA group treated with different doses of pectic polysaccharides from tamarillo pulp and receiving DSS in drinking water (STWA: 10, 30, or 100 mg/kg, [p.o.]). Weight loss, DAI, and colon length were measured at all groups. Specific dosing and measurements of oxidative stress, inflammatory, and histological parameters were only performed at a dose of 100 mg/kg STWA.

DAI was monitored daily and scored according to the following criteria: body weight: 0, if the animal’s body weight increased or remained within 1% of baseline; 1, if the animal’s body weight decreased by 1 to 5%; 2, if the animal’s body weight decreased by 5 to 10%; 3, if the animal’s body weight decreased by 10 to 15%; or 4, if the animal’s body weight decreased by more than 15%. Consistency of the stool: 0, no diarrhea; 2, the stool does not adhere to the animal’s anus; or 4, liquid stool. Presence of blood in the stool: 0, no blood in the stool; 2, presence of moderate blood; or 4, heavy bleeding.

At the end of the experimental protocol, the animals were euthanized, and the colon was carefully removed and washed with 0.9% saline. The tissue was then stored at −80 °C or fixed in formalin for later analysis. In addition, the animals’ feces were collected to measure occult blood.

### 4.5. Measurement of Occult Blood

During the experimental protocol, stool samples were collected on days 3, 5, and 8 of the protocol and examined for occult blood using the Meyer-Johannessen method [34]. For this purpose, the stool samples were diluted with 500 µL of water in a microtube. Then, 5 to 100 µL of Meyer-Johannessen reagent was added to the samples, followed by two to four drops of hydrogen peroxide. The presence of occult blood was indicated by an immediate color change to red. The symbols “+” and “−” were used to indicate the presence and absence of occult blood, respectively.

### 4.6. Preparation of the Tissue

To determine the parameters for oxidative stress and inflammation, the colon samples were homogenized in a phosphate-buffered saline solution (PBS) (pH 7.4) with protease inhibitor (Sigma FAST^TM,^ Sigma-Aldrich, Burlington, MA, USA). The resulting homogenate was used for the determination of reduced glutathione (GSH) and lipid peroxidation (LPO). The homogenate was then centrifuged at 9000 rpm at 4 °C for 20 min. The resulting supernatant was used for the determination of glutathione S-transferase (GST), superoxide dismutase (SOD), cytokine levels (TNF-α, IL-1β, and IL-10) and protein levels. The pellet was then resuspended in phosphate buffer containing hexadecyltrimethylammonium bromide (HTAB), as described below, and used to determine two specific markers: myeloperoxidase (MPO) and N-acetylglucosamine (NAG).

### 4.7. Quantification of the Proteins

Proteins were determined using the Bradford method [69]. In brief, 5 μL of the supernatant was added in duplicate to a 96-well plate. Then, 250 μL of the reaction solution (Bradford) was added to each well. The plate was incubated at 37 °C for 30 min and then the absorbance of the samples was measured using a spectrophotometer in the wavelength range of 540–590 nm.

### 4.8. Determination of the GSH Content

To determine the GSH content, 80 μL of tissue homogenate was added to a microtube containing 80 μL of 12.5% trichloroacetic acid (TCA). The microtubes were then vortexed and centrifuged at 9000× *g* for 15 min at 4 °C. After centrifugation, 20 µL of the supernatant (in duplicate) or a GSH standard curve was pipetted into a 96-well plate. For each well, 270 µL of Tris-HCl (400 mM, pH 8.9) and 10 µL of 5,5-dithiobis-2-nitrobenzoic acid (DTNB) (10 mM) solution were added. The plate was read at 415 nm on a spectrophotometer, and the absorbance values of the samples were interpolated using the GSH standard curve (6.25–400 µg/mL). The results were expressed as μg GSH/mg protein.

### 4.9. Determination of GST Activity

For the determination of GST activity, 200 μL of a reaction solution containing 0.1 M phosphate buffer, 1 mM 1-chloro-2,4-dinitrobenzene (CDNB), and 1 mM reduced glutathione (GSH) was mixed with 50 μL of sample supernatant. This mixture was run on a 96-well plate. The reaction was monitored for 3 min using a spectrophotometer at 340 nm. The GST activity was then calculated based on the extinction coefficient of 9.6/mM/cm^−1^. The GST activity results were expressed in nmol/min/mg protein.

### 4.10. Determination of SOD Activity

For the determination of SOD activity, aliquots of 20 μL of the supernatant were mixed with a buffer solution containing 442.5 μL Tris-HCl-EDTA (pH 8.5) and 25 μL pyrogallol. The samples were shaken and incubated for 30 min in the dark. The reaction was then stopped by adding 12.5 µL of 1 N HCl. The samples were centrifuged at 14,000 rpm for 4 min and transferred to a 96-well plate. The results were measured with a spectrophotometer at 405 nm and expressed in SOD units per milligram protein (U/mg protein).

### 4.11. Determination of LPO Levels

Aliquots of the colon homogenate were mixed with methanol at a ratio of 1:10 and centrifuged at 4 °C and 9000 rpm for 30 min to determine the LPO content. Then, 60 µL of the supernatant was pipetted into a microtube and mixed with 600 µL of FOX2 reaction (4 Mm butylated hydroxytoluene, 250 mM FeSO_4_, 25 mM H_2_SO_4_, and 100 mM orange xylenol). The samples were incubated for 30 min in the dark at room temperature and then centrifuged again for 30 min at 4 °C and 9000 rpm. The plate was read on a spectrophotometer at 560 nm. A standard curve of H_2_O_2_ (0.031–1000 μM) was used to calculate the result, and the result was expressed as μM H_2_O_2_/mg protein.

### 4.12. Quantification of MPO and NAG

First, the above pellets were resuspended with 1 mL of phosphate buffer (80 mM, pH 5.4) containing HTAB and sonicated for 1 min. Then, the samples were centrifuged at 11,000× *g* for 20 min at 4 °C. The supernatant was collected for analysis of MPO and NAG activity. For MPO determination, 30 μL of the sample supernatant was added to a 96-well plate together with 200 μL of a mixture of phosphate buffer (0.08 and 0.22 M) and 0.017% H_2_O_2_. Immediately afterward, the reaction started with 18.4 mM TMB (3,3′,5,5′-tetramethylbenzidine). The samples were incubated for 3 min at 37 °C. The reaction was then stopped by adding 30 μL of sodium acetate, and the absorbance was measured at 620 nm on a spectrophotometer.

To analyze the NAG activity, 25 μL of the supernatant was mixed with 25 μL of a solution containing NAG (4-nitrophenyl-N-acetyl-β-D-glucosaminide) and 100 μL of citrate buffer (50 mM, pH 4.5). The reaction was incubated at 37 °C for 60 min. Immediately afterward, the reaction was stopped with 100 μL glycine buffer (200 mM, pH 10.4), and the absorbance was immediately measured at 405 nm using a spectrophotometer. The results for both MPO and NAG were expressed as optical density (OD)/mg protein.

### 4.13. Evaluation of the Cytokine Levels

The supernatant of the samples was used to measure cytokine levels (IL-1β, TNF-α, and IL-10) using an enzyme-linked immunosorbent assay (ELISA) kit (PeproTech, Ribeirão Preto, SP, Brasil). Each cytokine level was determined by extrapolation from a standard curve. The results were expressed as pg of the respective cytokine/mg protein.

### 4.14. Histological Examination

The tissues removed for histological analysis were fixed with formalin, dehydrated, and embedded in paraffin. Paraffin blocks were then sectioned using a microtome with a thickness of either 5 μm or 7 μm. The resulting sections were mounted on glass slides, fixed, and stained with different dyes depending on the purpose of the analysis: Hematoxylin and Eosin (H&E) (to visualize tissue degradation and morphological changes in the samples), Periodic Acid Schiff (PAS), or Alcian blue (for neutral or acidic mucin staining, respectively).

### 4.15. Histopathological Score

H&E-stained sections were used to assess the histopathologic changes in the intestinal wall of the colon of mice. Histopathologic findings were classified into three categories based on previous studies: (i) presence and distribution of inflammatory infiltrates in the submucosa or intestinal mucosa; (ii) loss of intestinal mucosal architecture, including flattening of the mucosa, goblet cell depletion, epithelial erosion, ulceration, or abscess formation; and (iii) inflammatory infiltrates in the intestinal crypts (cryptitis). Histopathologic findings were graded according to severity from 0 to 3, with 0 being normal and 3 being severe. Ten microscopic fields per mouse were analyzed blindly using a light microscope (Leipzig Solstice 5Xi eLED, Leipzig, Germany) at 40× magnification (and 100× when necessary to confirm structures) [70,71].

### 4.16. Quantification of Goblet Cells

To quantify goblet cells producing neutral and acidic mucins, tissue sections were stained with periodic acid-Schiff (for neutral mucin-like glycoproteins) and Alcian blue (for acidic mucins). For this analysis, 10 images per mouse, from a total of 10 mice per group, were captured using a 10× objective and a high-resolution camera (Leipzig, HI-Speed, 2.0 megapixel) connected to a light microscope (Leipzig Solstice 5Xi eLED). The images were then transferred to a computer and processed using ImageJ^®^ software, version 1.53k. The quantification results were expressed as pixels per microscopic field [72].

### 4.17. Statistical Analysis

All data sets were checked for normality using the Shapiro–Wilk test prior to statistical analysis. In cases where the data had a parametric distribution, a one- or two-way analysis of variance (ANOVA) was performed, followed by a Bonferroni post hoc test for multivariate analyzes to compare differences between different groups. For data that did not have a parametric distribution, the Kruskal–Wallis test and then Dunn’s post hoc test were used. Parametric data were presented as means ± SEM, while non-parametric data were presented as medians and interquartile ranges. Differences with a *p*-value < 0.05 were considered statistically significant. All statistical analyses were performed using GraphPad Prism software, version 8.0.

## 5. Conclusions

The results suggest that STWA may have a protective effect against ulcerative colitis in mice. Treatment with STWA (100 mg/kg) effectively prevented weight loss, reduced disease activity, and preserved colon length. It also alleviated oxidative stress by increasing glutathione S-transferase levels and decreasing lipid peroxidation. In addition, STWA showed anti-inflammatory effects by decreasing myeloperoxidase, N-acetyl-β-glucosaminidase, and TNF-α levels while increasing IL-10. Histologic analysis confirmed preservation of the colonic epithelium, indicating less tissue damage. Although our study has limitations, such as the lack of more comprehensive tests to elucidate the exact mechanism of action and evaluate specific toxicity parameters, these results emphasize the potential of tamarillo polysaccharides as a natural therapeutic approach for ulcerative colitis. Nevertheless, further studies are needed to clarify their mechanisms of action and long-term effects.

## Figures and Tables

**Table 1:** Evaluation of occult blood in mouse stools.

Animal	C	DSS	STWA (10 mg/kg)	STWA (30 mg/kg)	STWA (100 mg/kg)
1	−	+	+	−	−
2	−	+	+	−	−
3	−	+	−	+	+
4	−	+	+	+	−
5	−	+	+	−	+
6	−	+	−	+	−

(+) Positive for occult blood; (−) negative for occult blood. n = 6 animals per group.

## Data Availability

The original contributions presented in this study are included in the article. Further inquiries can be directed to the corresponding author.
